# Osteogenic differentiation of fibroblast-like synovial cells in rheumatoid arthritis is induced by microRNA-218 through a ROBO/Slit pathway

**DOI:** 10.1186/s13075-018-1703-z

**Published:** 2018-08-29

**Authors:** Naoki Iwamoto, Shoichi Fukui, Ayuko Takatani, Toshimasa Shimizu, Masataka Umeda, Ayako Nishino, Takashi Igawa, Tomohiro Koga, Shin-ya Kawashiri, Kunihiro Ichinose, Mami Tmai, Hideki Nakamura, Tomoki Origuchi, Ko Chiba, Makoto Osaki, Astrid Jüngel, Steffen Gay, Atsushi Kawakami

**Affiliations:** 1 Department of Immunology and Rheumatology, Division of Advanced Preventive Medical Sciences, Nagasaki University Graduate School of Biomedical Sciences, 1-7-1 Sakamoto, Nagasaki, 852-8501 Japan; 2 Medical Education Development Center, Nagasaki University School Hospital, Nagasaki, Japan; 3 Center for Comprehensive Community Care Education, Nagasaki University Graduate School of Biomedical Sciences, Nagasaki, Japan; 4 Center for Bioinformatics and Molecular Medicine, Nagasaki University Graduate School of Biomedical Sciences, Nagasaki, Japan; 5 Departments of Community Medicine, Division of Advanced Preventive Medical Sciences, Nagasaki University Graduate School of Biomedical Sciences, Nagasaki, Japan; 6 Department of Physical Therapy, Nagasaki University Graduate School of Biomedical Sciences, Nagasaki, Japan; 7 Department of Orthopedic Surgery, Nagasaki University Graduate School of Biomedical Sciences, Nagasaki, Japan; 8 Center of Experimental Rheumatology, University Hospital Zurich and University of Zurich, Schlieren, Zurich, Switzerland

**Keywords:** miR-218, Rheumatoid arthritis, Fibroblast-like synovial cells, Osteoblast

## Abstract

**Background:**

Fibroblast-like synovial cells (FLS) have multilineage differentiation potential including osteoblasts. We aimed to investigate the role of microRNAs during the osteogenic differentiation of rheumatoid arthritis (RA)-FLS.

**Methods:**

RA-FLS were differentiated in osteogenic medium for 21 days. Osteogenic differentiation was evaluated by alkaline phosphatase (ALP) staining and Alizarin Red staining. MicroRNA (miRNA) array analysis was performed to investigate the differentially expressed miRNAs during osteogenic differentiation. Expression of miR-218-5p (miR-218) during the osteogenic differentiation was determined by quantitative real-time PCR. Transfections with an miR-218 precursor and inhibitor were used to confirm the targets of miR-218 and to analyze the ability of miR-218 to induce osteogenic differentiation. Secreted Dickkopf-1 (DKK1) from FLS transfected with miR-218 precursor/inhibitor or roundabout 1 (ROBO1) knockdown FLS established using ROBO1-small interfering RNA (siRNA) were measured by ELISA.

**Results:**

The miRNA array revealed that 12 miRNAs were upregulated and 24 miRNAs were downregulated after osteogenic differentiation. We observed that the level of miR-218 rose in the early phase of osteogenic differentiation and then decreased. Pro-inflammatory cytokines modified the expression of miR-218. The induction of miR-218 in RA-FLS decreased ROBO1 expression, and promoted osteogenic differentiation. Both the overexpression of miR-218 and the knockdown of ROBO1 in RA-FLS decreased DKK1 secretion.

**Conclusion:**

We identified miR-218 as a crucial inducer of the osteogenic differentiation of RA-FLS. MiR-218 modulates the osteogenic differentiation of RA-FLS through the ROBO1/DKK-1 axis. The induction of the osteogenic differentiation of proliferating RA-FLS through the provision of miR-218 into RA-FLS or by boosting the cellular reservoir of miR-218 might thus become a therapeutic strategy for RA.

**Electronic supplementary material:**

The online version of this article (10.1186/s13075-018-1703-z) contains supplementary material, which is available to authorized users.

## Background

Rheumatoid arthritis (RA) is a chronic inflammatory disease characterized by marked hyperplasia of the lining layer of the synovium, leading to the destruction of articular cartilage and bone. In RA pathogenesis, fibroblast-like synovial cells (FLS) are pivotal. FLS contribute to the production of pro-inflammatory cytokines, small molecule mediators of inflammation, and proteolytic enzymes that degrade the extracellular matrix [1]. Moreover, FLSs are resistant to programmed cell death [2], resulting in an aggressive, invasive phenotype similar to that of an invasive cancer, and the hyperplastic synovial tissue, also called the pannus, destroys cartilage and bone. Although rapid development of cytokine-targeted therapeutic agents such as tumor necrosis factor (TNF) inhibitors has provided better clinical outcomes including achievement of remission for patients with RA, there are many unfavorable problems such as inadequate response, high cost, and adverse events such as infections [3, 4]. RA-FLS-targeted therapies have thus been explored, and several key mediators that activate cytokine production from FLS [5, 6] or acquire anti-apoptosis property [7] had been elucidated. However, despite the enthusiasm for developing new treatments that directly target FLS, no directly FLS-targeted therapy is available at this time.

In RA, FLS of mesenchymal origin conserve mesenchymal properties. The gene expression pattern of FLS is similar to that of mesenchymal stem cells [8], and in vitro studies have shown that appropriate stimulation in culture induces differentiation of FLS into chondrocytes, adipocytes, muscle cells, and osteoblasts [9–11]. FLS are bone marrow (BM)-derived mesenchymal cells (MSCs) [12], and the multi-linage potential of arthritic FLS is thought to be arrested at the early stage of differentiation by activation of nuclear factor-κB (NF-κB) [13]. Forced cell differentiation might become a candidate therapeutic option for RA; for example, mesenchymal stromal cells showed reduced interleukin-6 (IL-6) production after their differentiation into adipocytes [14]. Until now, there has been no evidence that FLS differentiate into osteoblasts in joints. However, if the induction of intrinsic transdifferentiation of markedly proliferating FLS in the joints causes differentiation of FLS into osteoblasts, it might become a treatment option for RA.

MicroRNAs (miRNAs) are small non-coding RNAs, which regulate gene expression post-transcriptionally by binding to the 5′ untranslated region (UTR), coding regions or 3′ UTR of mRNA [15]. Altered expression of miRNAs has been reported in many diseases such as cancer, infections, and autoimmune diseases including RA, and this might arise from a modulation of diverse biological processes such as cell proliferation, apoptosis, metabolism and cell differentiation by miRNAs [16–18]. There is growing evidence that miRNAs are critical in osteoblast differentiation [19].

Jie et al. reported that miR-145 suppressed the osteogenic differentiation of mouse osteoblastic and myoblastic cell lines (MC3T3 and C2C12) by targeting Sp7 [20]. Other reports revealed that several miRs, e.g., miR-218, miR-34 and miR-195 modulate osteogenic differentiation by suppressing their targets [21–23]. However, the effect of miRNAs on RA-FLS differentiation including osteoblast differentiation had not yet been elucidated.

In the present study, we identified a miRNA (miR-218) that was altered during the osteogenic differentiation of RA-FLS, and we confirmed that this miRNA indeed promoted the osteogenic differentiation of RA-FLS. Our findings also revealed that Wnt/β-catenin signaling is involved in the promotion of the osteogenesis of RA-FLS by miR-218.

## Methods

### Isolation of FLS and stimulation assays

We obtained synovial tissues from patients with RA who fulfilled the 2010 American College of Rheumatology (ACR)/European League Against Rheumatism (EULAR) classification criteria for RA [24] or the 1987 ACR classification criteria for RA [25] at the time of orthopedic surgery. Each patient provided a signed consent form to participate in the study, which was approved by the Institutional Review Board of Nagasaki University and the Swiss Ethical commission. FLS were isolated from synovial tissues as described previously [26, 27]. Cells were cultivated in Dulbecco’s modified Eagle’s medium (DMEM) containing 10% heat-inactivated fetal bovine serum (FBS), 100 units/ml penicillin and 100 ng/ml streptomycin (all from Gibco, Basel, Switzerland). FLS from passages 3–8 in monolayer culture were used for the experiments. In the stimulation experiments, FLS were stimulated for 24 h with recombinant TNF-α (10 ng/ml), interleukin-1β (IL-1β) (1 ng/ml) (R&D Systems, Abingdon, UK), recombinant interleukin-6 (IL-6) (100 ng/ml) and recombinant soluble IL-6 receptor (sIL-6R) (100 ng/ml) (Peprotech, Rocky Hill, NJ, USA).

### Osteogenic differentiation in vitro

RA-FLSs were plated at a cell density of 1 × 10^4^ in 12-well plates. After they were 70% confluent, medium was replaced with osteogenic differentiation Bulletkit™ medium containing dexamethasone, ascorbate, glycerophosphate, L-Glutamine, Pen/Strep and MCGS (Lonza, Walkersville, MD, USA) to differentiate to osteoblasts. RA-FLS was cultured in the induction medium for up to 21 days. The medium was changed every 3 days. Osteoblast differentiation was evaluated by alkaline phosphatase (ALP) staining and Alizarin Red staining.

### ALP staining and Alizarin Red staining

After the osteogenic induction or transfection experiments, cells were fixed in 4% paraformaldehyde and stained with ALP using ALP staining kit (Cosmo Bio, Tokyo). In another set of experiments, we performed Alizarin Red staining to detect the calcification after 21 days of culture in induction medium (late period of induction). Cells were fixed in methanol and stained with Alizarin Red using Calcified Nodule Staining kit (Cosmo Bio). ALP-positive cells were stained blue by ALP staining, and calcium nodules were detected as red bodies by Alizarin Red staining.

### Transfection experiments

For a transient transfection approach with the aim to inhibit or enhance the miR-218 function, RA-FLSs were transfected with synthetic precursor miRNA (Pre-miR), with inhibitors of miR-218 (anti-miR), or with scrambled controls (Pre-miR/Anti-miR Negative Control #1; Ambion/Applied Biosystems, Foster City, CA, USA) at a final concentration of 100 nM with the use of Lipofectamine 2000 reagent (Invitrogen, Carlsbad, CA, USA). In another set of experiments, RA-FLSs were transfected with specific small interfering RNA (siRNA) that target ROBO1 mRNA using FlexiTube siRNA Premix (Qiagen, Hilden, Germany) at a final concentration of 25 nM according to the manufacturer’s protocol. AllStars Negative control siRNA (siRNA-premix, Qiagen) served as a control. Transfection efficiency of pre/anti-mir-218 and siRNA were controlled by TaqMan-based real-time polymerase chain reaction (PCR).

### RNA isolation and quantitative real-time PCR analysis

A *mir*Vana miRNA Isolation kit was used for isolation of total RNA (Ambion/Applied Biosystems). Specific single TaqMan miRNA assays (Ambion/Applied Biosystems) were used to measure the expression levels of selected miRNA in a model light cycler 1.5 (Roche Diagnostics). Expression of the U6B small nuclear RNA (RNU6B) was used as endogenous control to normalize the data. In the analysis of the expression of specific mRNA, gene expression was quantified using SYBR Green Real-time PCR, as previously described [27]. The primers were obtained from Takara Bio (Tokyo) and Integrated DNA Technologies (Coraville, IA, USA). The primer sequences are shown in Table 1. The amounts of loaded complementary DNA (cDNA) were normalized using glyceraldehyde-3-phosphate dehydrogenase (GAPDH) as an endogenous control. For relative quantification, the comparative threshold cycle (Ct) method was used.

**Table 1:** SYBR green primers used for real-time PCR

ALP forward	5′-AGCTCAACACCAACGTGGCTAA-3′
ALP reverse	5′-TTGTCCATCTCCAGCCTGGTC-3′
RUNX2 forward	5′-CTTTGTAGCACAAACATTGCTGGA-3′
RUNX2 reverse	5′-AAAGCTGTGGTACCTGTTCTGGA-3′
CTNNB1 forward	5′-CATCCTAGCTCGGGATGTTCAC-3′
CTNNB1 reverse	5′-TCCTTGTCCTGAGCAAGTTCAC-3′
CDH11 forward	5′-CAGGTGCTACAGCGCTCCAA-3′
CDH11 reverse	5′-TTAATGTTCCCATCACCAGAGTCAA-3′
ROBO1 forward	5′-CGGCAGAGTATGCTGGTCTGAA-3′
ROBO1 reverse	5′-CTAGGGCACTGAGACGCATGAA-3′
DKK1 forward	5′-CCAGACCATTGACAACTACCAG-3′
DKK1 reverse	5′-AGGCGAGACAGATTTGCAC-3′
GAPDH forward	5′-GCACCGTCAAGGCTGAGAAC-3′
GAPDH reverse	5′-TGGTGAAGACGCCAGTGGA-3′

*CDH11* cadherin 11, *ALP* alkaline phosphatase, *RUNX2* runt related transcription factor 2, *CTNNB* catenin beta 1, *ROBO1* roundabout 1, DKK1 dickkopf-1, *GAPDH* glyceraldehyde-3-phosphate dehydrogenase

### In silico prediction analysis of miRNA targeting genes

MiRecords (http://c1.accurascience.com/miRecords/) was used to predict miRNA transcript targets. MiRecords is a database that combines the following miRNA target prediction tools: DIANA-microT, Micro inspector, miRanda, Mir Target2, mi Target, NB miRTar, Pic Tar, PITA, RNA hybrid, and TargetScan. Results were filtered based on the observation that a given miRNA targeted a transcript present in a minimum of five of these miRNA target prediction tools.

### ELISA

Protein in cell supernatant was detected by ELISA with an ELISA kit specific for Dickkopf-1 (DKK1) according to the manufacturer’s instructions (R&D Systems). Absorption was measured at 450 nm.

### miRNA and DNA microarray assay analyses

miRNA expression profiles during osteogenic differentiation were established by applying SurePrint G3 Human miRNA, 8 × 60 K (release 18.0) microarrays containing 1887 human miRNA oligonucleotide probes (Agilent Technologies, Santa Clara, CA, USA). DNA microarray analysis was performed using whole human genome DNA microarray SurePrint G3 Human Gene Expression, 8 × 60 K (ver. 2) microarrays (Agilent Technologies). All procedures were carried out according to the manufacturer’s recommendations. Microarray data were analyzed by GeneSpring software ver. 12.5.0 or 12.6.1 (Agilent Technologies). The raw signals were log2 transformed and normalized using the percentile shift normalization method: the value was set at the 90th percentile for miRNA microarray and the 75th percentile for DNA microarray.

### Statistical analyses

GraphPad Prism software (GraphPad, San Diego, CA, USA) was used for statistical analyses. Normal distributions of the data were confirmed using the Kolmogorov-Smirnov test. Statistical significance was evaluated by Student’s paired *t* test (for parametric data) or the Wilcoxon matched-pairs signed rank test (non-parametric data) for related data. All data are expressed as the mean ± standard error of the mean (SEM). *p* values < 0.05 were considered significant.

## Results

### RA-FLS osteogenic differentiation

**Fig. 1:**
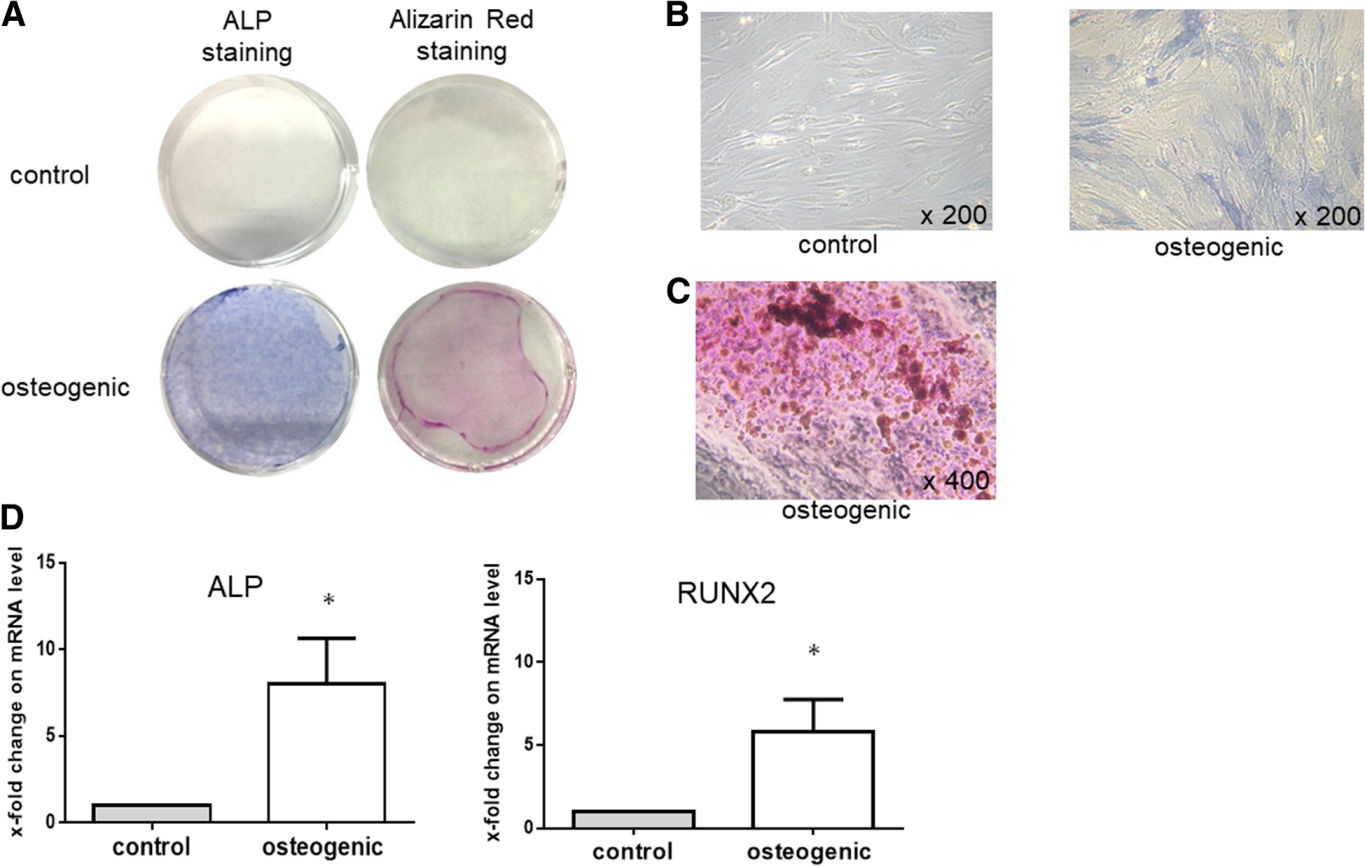
Osteogenic differentiation of rheumatoid arthritis (RA)-fibroblast-like synovial cells (FLS) cultured in osteogenic induction medium. **a** Representative images of alkaline phosphatase (ALP) staining (right) and Alizarin Red staining (left). RA-FLS (*n* = 6) were cultured in osteogenic induction medium or control medium. After 21 days, cells were stained with ALP and Alizarin Red. **b** Representative image of the morphology of RA-FLS with ALP staining. Right: RA-FLS were cultured in control medium. Left: RA-FLS were cultured in osteogenic induction medium (magnification × 200). **c** Visualization of calcified nodules by Alizarin Red staining. RA-FLS were cultured in osteogenic induction medium (magnification × 400). **d** RA-FLS (*n* = 5) were cultured in osteogenic induction medium or control medium for 21 days. Expression of ALP, runt related transcription factor 2 (RUNX2) was determined by quantitative real-time PCR. Values are presented as means ± SEM: *p < 0.05 versus control, as determined by Student’s paired *t* test or Wilcoxon matched-pairs signed rank test

We first investigated whether RA-FLS can differentiate into osteoblasts. To induce the differentiation of RA-FLS, the medium was replaced with osteogenic induction medium. Osteoblasts were then evidenced by ALP staining (Fig. 1a, b) and Alizarin Red staining for matrix mineralization (Fig. 1a, c). ALP and runt related transcription factor 2 (RUNX2) were used as phenotypic markers of osteogenic differentiation, and as shown in Fig. 1d, the expression of those mRNAs was significantly increased at day 21 after osteogenic induction.

### Expression of miR-218 during osteogenic differentiation of RA-FLS

To explore miRNAs involved in the osteogenic differentiation of RA-FLS, we started by examining the miRNA expression profiles by microarrays. Our microarray analysis of three pairs (RA-FLS cultured in osteogenic induction medium versus an untreated control) identified 36 differentially expressed miRNAs among all three pairs. Of these, 12 miRNAs were upregulated miRNA s and 24 were downregulated miRNAs (Additional file 1: Figure S1).

**Fig. 2:**
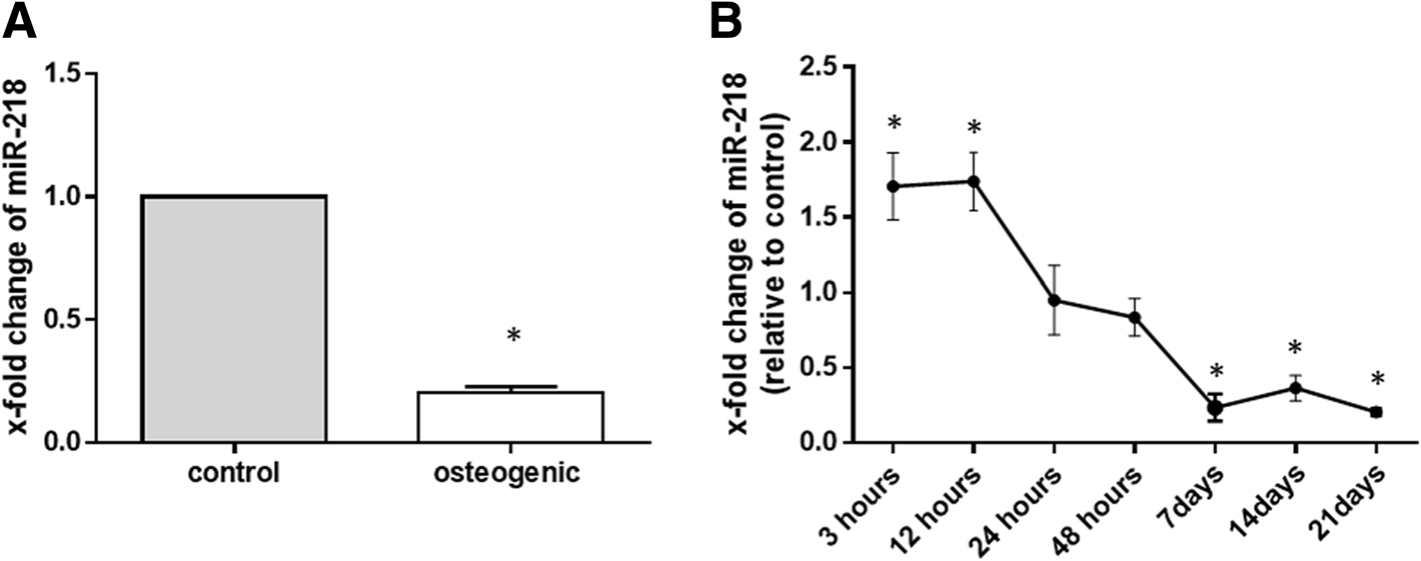
Expression of microRNA-218-5p (miR-218) during osteogenic differentiation of rheumatoid arthritis (RA)-fibroblast-like synovial cells (FLS), as determined by TaqMan-based Real-time polymerase chain reaction analysis. Expression of miR-218 in osteogenic differentiation was determined relative to the controls, which was defined as 1. **a** miR-218 was markedly reduced in RA-FLS (*n* = 5) at 21 days after culture in osteogenic induction medium compared to culture in control medium. Values are presented as means ± SEM: *p < 0.0001 versus control, as determined by Student’s paired *t* test. **b** The time course of expression of miR-218 during osteogenic differentiation (*n* = 4–5). Points and bars represent means and SEM respectively: *p < 0.05 versus control, as determined by Student’s paired *t* test

Among these miRNAs, miR-218-5p (miR-218) was one of the most significantly and consistently downregulated miRNAs after osteogenic induction. To validate the microarray findings, quantitative real-time PCR with additional RA-FLS cultured in osteogenic induction medium was performed. In agreement with the microarray analysis results, there was significant downregulation in the expression of mir-218 after osteogenic induction. The miR-218 decreased with fold-change of 0.203 ± 0.026 (*p* < 0.0001) at day 21 after osteogenic induction compared with the untreated control (Fig. 2a). We next investigated the time course of miR-218 expression during osteogenic differentiation. In response to osteogenic differentiation, miR-218 rose until 12 h and then decreased at 7 days, and remained decreased at 21 days (Fig. 2b).

### miR-218 promotes osteogenesis of RA-FLS

**Fig. 3:**
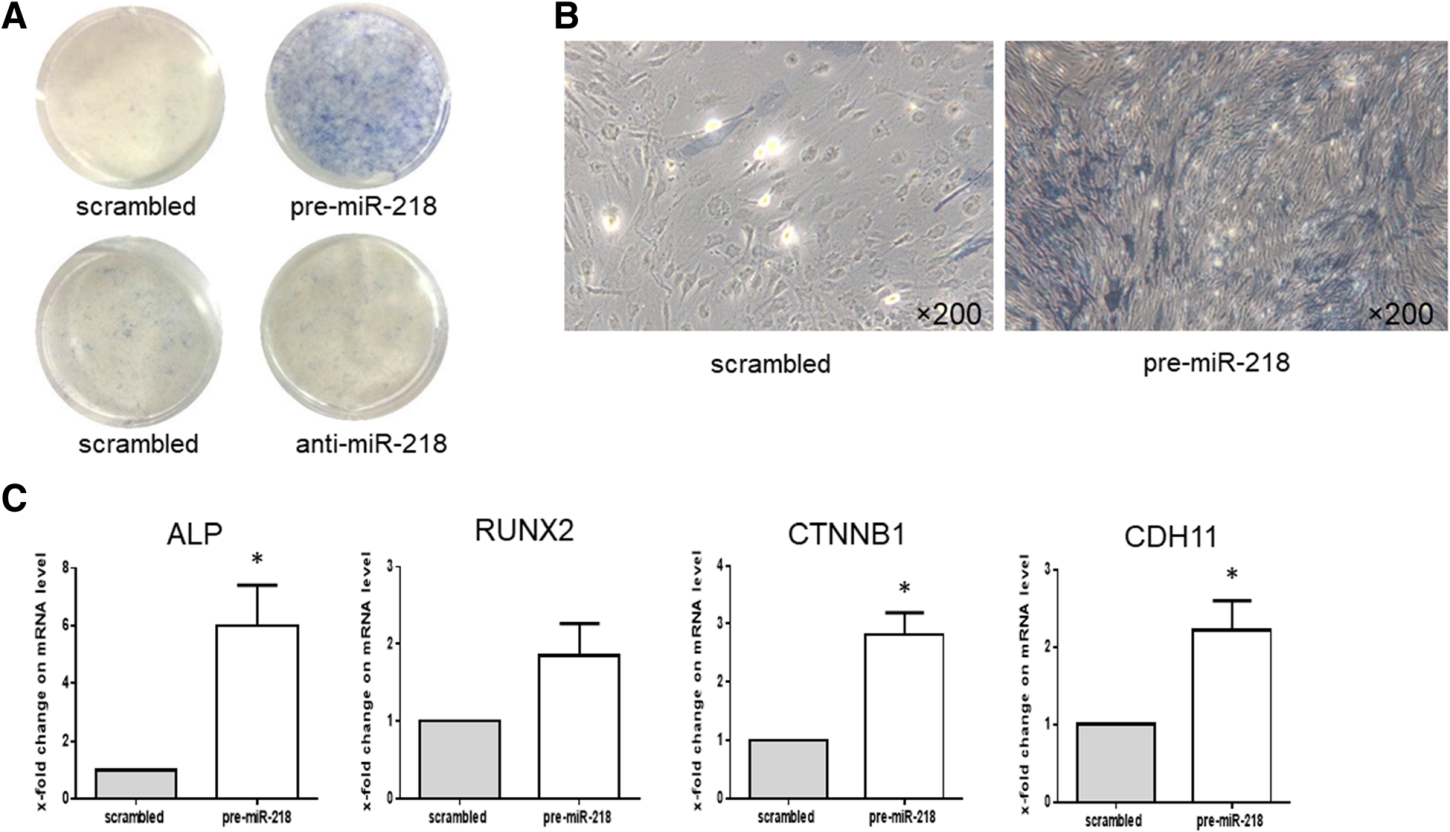
Overexpression of microRNA-218-5p (miR-218) promotes osteogenic differentiation of rheumatoid arthritis (RA)-fibroblast-like synovial cells (FLS). **a** Alkaline phosphatase (ALP) staining at day 14 showed the enhanced ALP activity of osteogenic differentiation after transfection with precursor miR-218 (pre-miR-218) compared to scrambled RNA-transfected controls. Images are representative of five samples. **b** The morphology of RA-FLS with ALP staining. Right: RA-FLS were transfected with scrambled control. Left: RA-FLS were transfected with pre-miR-218 (magnification × 200). Images are representative of five samples. **c** Transfection of RA-FLS (*n* = 5) with pre-miR-218 for 72 h compared with scrambled RNA transfected controls increased the level of ALP, runt related transcription factor 2 (RUNX2), catenin beta 1 (CTNNB1), cadherin 11 (CDH11) as determined by quantitative real-time PCR analysis. Values are presented as means ± SEM: *p < 0.05 versus scrambled RNA-transfected controls, as determined by Student’s paired *t* test

To determine the role of miR-218 in the osteogenic differentiation of RA-FLS, RA-FLS were transfected with pre-miR-218 or anti-miR-218 and the respective negative control. In RA-FLS, transfection with pre-miR-218 increased the levels of miR-218 with fold-change of 1.34 × 10^5^ ± 1.70 × 10^5^ compared to the scrambled control. Knockdown with anti-miR-218 reduced the expression of miR-218 with fold-change of 0.098 ± 0.058 indicating successful transfection. At 14 days after transfection, osteogenesis ability was examined. Strong ALP staining was observed (Fig. 3a, b). Interestingly, in addition to ALP and RUNX2 mRNA, other osteogenesis-associated genes such as catenin beta 1 (CTNNB1) and cadherin 11 (CDH11) were also significantly upregulated in pre-miR-218 transfected RA-FLS compared to the scrambled control from 72 h after transfection (Fig. 3c). In contrast, in the anti-miR-218 transfected RA-FLS, no ALP staining was observed (Fig. 3a), and osteogenic specific markers were not upregulated. Moreover, the transfection of anti-miR-218 did not attenuate the osteogenic differentiation induced by osteogenic induction medium (please contact author for data requests). This gain-and-loss of function assays with miR-218 showed miR-218 solely induced the osteogenic differentiation of RA-FLS without osteogenic induction medium.

### The expression of miR-218 is modulated by pro-inflammatory cytokines

We next investigated whether miR-218 is modulated in the physiopathological condition of RA. To simulate the inflammatory conditions present in RA in vivo, we stimulated RA-FLS with TNF-α and IL-1β or IL-6. Although the difference was not statistically significant, the stimulation of these cytokines downregulated the expression of miR-218 numerically (Additional file 2: Figure S2). This result suggests that the conditions in which inflammation occurs in RA, which is a bone erosion-progressive state, also presents a disadvantage for the osteogenic differentiation of RA-FLS.

### ROBO1 is targeted by miR-218 in RA-FLS

**Fig. 4:**
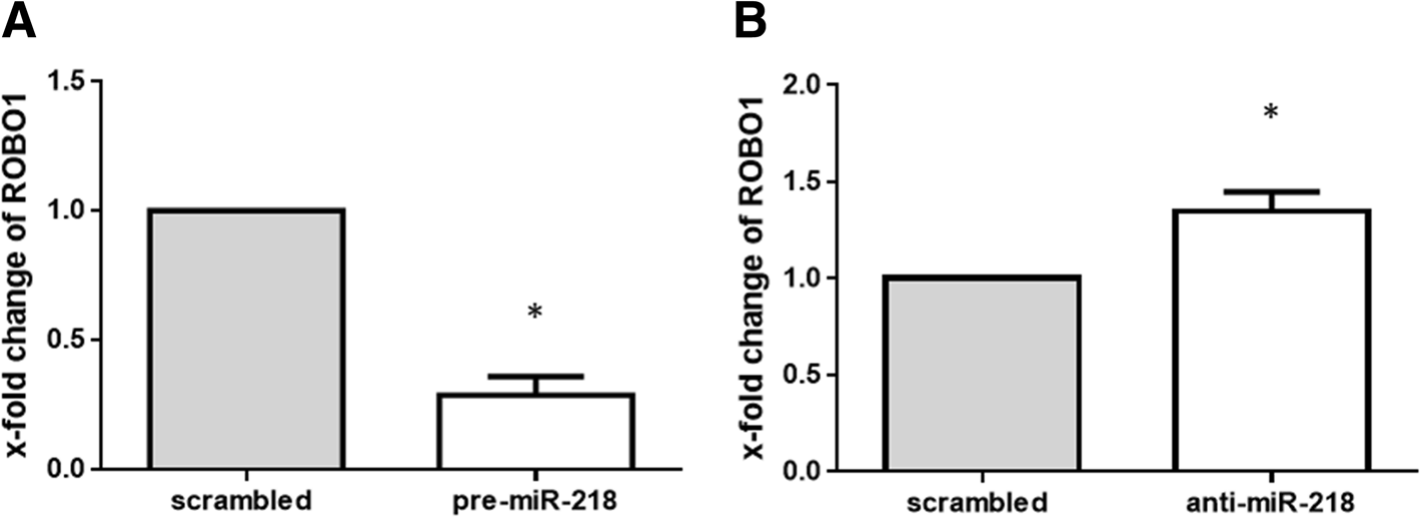
Influence of overexpression and knockdown of microRNA-218-5p (miR-218) on the expression of roundabout1 (ROBO1). Expression of ROBO1 in rheumatoid arthritis (RA)-fibroblast-like synovial cells (FLS) was determined relative to the controls transfected with scrambled RNA, which was defined as 1. **a** Transfection of RA-FLS (*n* = 5) with precursor miR-218 (pre-miR-218) for 48 h decreased the levels of ROBO1 compared to scrambled RNA-transfected controls, as determined by SYBR green real-time PCR analysis. **b** Knockdown of miR-218 for 48 h in RA-FLS (*n* = 5) increased the level of ROBO1 compared to scrambled RNA-transfected controls, as determined by SYBR green real-time PCR analysis. Values are presented as means ± SEM: *p < 0.05 versus scrambled RNA-transfected controls, as determined by Student’s paired *t* test

To elucidate the functional consequences of upregulation of miR-218, we searched for potential gene targets of miR-218 that might contribute to the osteogenesis of RA-FLS. We performed a DNA microarray using gain-and-loss of function assays with miR-218, and we also conducted an in silico identification of potential gene targets of miR-218 using the MiRecords (http://c1.accurascience.com/miRecords/). From the microarray result, the genes increased by knockdown of miR-218 and decreased by overexpression of miR-218 were considered as potential targets (microarray data are available from Gene Expression Omnibus (GEO, http://www.ncbi.nlm.nih.gov/geo/) [GEO:GSE 111946]. Among the candidates that were predicted by both in silico and microarray analyses, we focused on roundabout 1 (ROBO1), a transmembrane receptor proteins implicated in the Slit-ROBO pathway with an established relationship to osteogenesis [28], and we further analyzed ROBO1. Overexpression of miR-218 reduced the expression of ROBO1 with fold-change of 0.29 ± 0.07 (*p* < 0.05) at the mRNA level. Conversely, knockdown of miR-218 increased the expression of ROBO1 with fold-change of 1.34 ± 0.10 (*p* < 0.05) (Fig. 4a, b). Taken together, these findings confirmed ROBO1 as a target of miR-218 in RA-FLS.

### miR-218 and the suppression of ROBO1 promote osteogenesis through DKK1 suppression

**Fig. 5:**
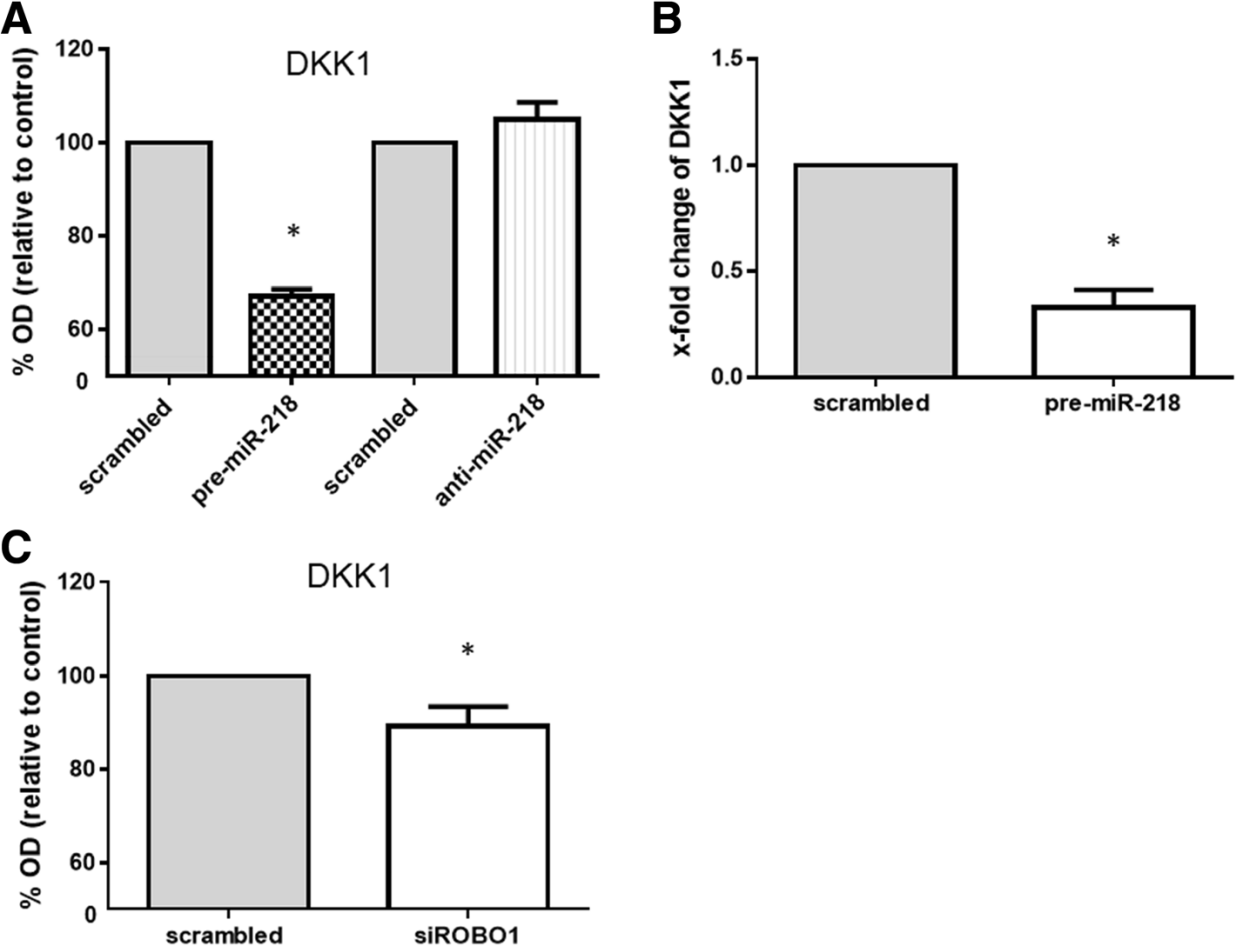
Suppression of Dickkopf-1 (DKK1) by overexpression of microRNA-218-5p (miR-218) or silencing of roundabout1 (ROBO1). **a** At the protein level, transfection of rheumatoid arthritis (RA)-fibroblast-like synovial cells (FLS) (*n* = 4) with precursor miR-218 (pre-miR-218) for 48 h decreased DKK1 protein production in the culture supernatant compared to scrambled RNA transfected controls, as determined by ELISA. Graphs represent optical density (OD) value; each mean amount of DDK1 protein are as follows; scrambled control: pre-miR-218 1.17 ng/ml:0.76 ng/ml, scrambled control: anti-miR-218 1.58 ng/ml:1.65 ng/ml, respectively. **b** At the mRNA level, transfection of RA-FLS (*n* = 4) with pre-miR-218 for 48 h decreased DKK1 expression compared to scrambled RNA transfected controls, as determined by SYBR green real-time PCR analysis. **c** Secretion of DKK1 from RA-FLS (*n* = 4) was decreased after transfection with ROBO1-specific small interfering RNA (siRNA) compared to the scrambled RNA transfected controls, as determined by ELISA. Graphs represent OD value; each mean amount of DDK1 protein are as follows; scrambled control: siROBO1.28 ng/ml:1.09 ng/ml, respectively. Values are presented as means ± SEM: *p < 0.05 versus scrambled RNA-transfected controls, as determined by Student’s paired *t* test

Wnt/β-catenin signaling plays a crucial role in osteogenesis [29], therefore we next investigated whether miR-218 affects inhibitor of Wnt/β-catenin signaling. The level of DKK1 (which has been shown to be a potent inhibitor of Wnt/β-catenin signaling) that we detected in RA-FLS transfected with pre-miR-218-conditioned medium was significantly reduced compared to that detected in scrambled control-conditioned medium (Fig. 5a). These findings were confirmed at the mRNA level by quantitative real-time PCR. Overexpression of miR-218 reduced the expression of DKK-1 with fold-change of 0.33 ± 0.08 (*p* < 0.005) (Fig. 5b).

To mimic the promotion of the osteogenic condition of RA-FLS by miR-218 as we observed, we silenced the expression of ROBO1 with siRNA. After transfection with ROBO1-specific siRNA, the expression of ROBO1 was decreased with fold-change of 0.30 ± 0.08, indicating successful transfection. Similar to the effect of miR-218 overexpression, silencing of ROBO1 reduced DKK1 secretion from RA-FLS (Fig. 5c). These results suggest that miR-218 promote osteogenic differentiation of RA-FLS through ROBO1 suppression and inhibition of DKK1 secretion, therefore activation of Wnt/β-catenin signaling is presumed to be the possible mechanism of miR-218-induced osteogenesis of RA-FLS.

## Discussion

This is the first study to show that a miRNA could induce the osteogenic differentiation of FLS from RA, a bone-erosive disease. Our findings demonstrated that the expression of miR-218 was altered during osteogenic induction and most interestingly, miR-218 directly promoted the osteogenic differentiation of RA-FLS through the suppression of DKK1.

Skeletal homeostasis is a continuous process that is maintained by a balance between bone resorption by osteoclasts and bone formation by osteoblasts. In RA, bone erosion is considered to be the result of a disruption of this balance, inadequate bone formation, and an enhancement of osteoclast activity [30]. Inadequate bone formation in RA was recently elucidated. Two studies reported that IL-6, a key pro-inflammatory cytokine of RA, decreased osteoblast proliferation and induced osteoblast apoptosis [31, 32]. IL-6 inhibited the formation of mineralized bone nodules in an in vitro rat osteogenesis model [32]. Another study focused on DKK1, which we observed to be a key regulator of the promotion of osteogenesis by miR-218. DKK1 expression was increased in FLS and endothelial cells in an animal model of arthritis, and TNF markedly increased the production of DKK1 from cultured FLS. In addition, serum DKK1 was elevated in patients with RA [33].

An in vivo study by Walsh et al. using an animal model of RA demonstrated that the presence of inflammation modified osteoblast-lineage cell function, resulting in impaired osteoblast maturation and significant reduction of mineralized bone formation within the site of arthritic erosion [34]. In clinical practice, the repair of bone erosion is uncommon but it has been demonstrated to occur. For example, 6% of patients with RA treated with adalimumab were shown to have bone repair [35], and 1-year treatment with TNF inhibitor was shown to reduce the mean depth of erosion detected by high-resolution computed tomography [36]. Although the mechanisms underlying the repair of bone erosion in RA have been not elucidated, the possible main mechanism might be the correction of the imbalance of bone remodeling that arises from inflammation. It is not elucidated that the osteogenic differentiation of RA-FLS, which we showed in an in vitro study, occurs in the joints in RA. However, if it does occur in the joints in RA, it is possible that proliferation of FLS contributes to bone repair by induction of osteogenic differentiation by miR-218.

Although the role of miR-218 in human disease and cell physiology has not been widely addressed, several studies of miR-218 have been reported. For example, miR-218 suppresses gastric cancer cell proliferation via regulation of angiopoietin-2 [37], and miR-218 inhibits proliferation of glioma cells by targeting ROBO1 [38]. Two studies revealed that miR-218 promotes osteogenic differentiation of mesenchymal stem cells through regulation of Wnt/β-catenin signaling, targeting DKK2, sclerostin, and secreted frizzled related protein 2 [22, 39]. The difference in targets compared to our present study might be due to the difference in the types of cells examined, because miRNA may have different effects depending on cell type.

Our study suggests that the ROBO1-DKK1 axis is important for osteogenesis in RA-FLS. ROBO1 is a member of the ROBO family; it serves as a transmembrane receptor of Slit, and emerging evidence has indicated that a ROBO/Slit signaling pathway is crucial in axon guidance [40]. In addition to axon guidance, the ROBO/Slit pathway is also involved in cell processes such as cell proliferation, cell motility, and angiogenesis [41, 42]. The effect of the ROBO/Slit signaling pathway in osteogenesis remains unknown, but Sun et al. reported that slit2 reduced ALP expression and osteoblastic gene expression in the osteoblastic cell line MC3T3-E1 [28]. Our present findings also showed that knockdown of ROBO1 significantly reduced DKK1 secretion from RA-FLS.

Wnt/β-catenin signaling is known as one of the important molecular cascades and is central to osteogenesis, and DKK1 is a potent inhibitor of this signaling pathway, causing deregulation of bone formation [43]. As described above, in vivo and in vitro studies have shown an increase of DKK1 in both an arthritic animal model and in patients with RA. In fact, patients with RA with radiological progression within 2 years have been shown to have higher baseline levels of serum DKK1 compared to the patients without radiological progression [44]. Wang et al. reported that serum DKK1 is significantly correlated with bone erosion, and that treatment with a TNF-α inhibitor or IL-1 receptor antagonist decreased serum DKK1 levels [45]. Considering these results, the reduction of DKK1 secretion by miR-218 might provide a protective effect against RA bone erosion besides the effect of miR218 toward RA-FLS osteogenesis.

In the present study, miR-218 promoted osteogenic differentiation despite a significant decrement of miR-218 after osteoblast differentiation. A negative and positive feedback loop between microRNA and its target gene or cellular response have been observed [46–48]. This crosstalk was also seen in the Wnt/β-catenin signaling pathway; miR-122 inhibits the Wnt/β-catenin signaling pathway, which negatively regulates the expression of miR-121 in glioma cells [49]. miR-372 and miR-373 activate the Wnt/β-catenin signaling pathway by targeting Wnt/β-catenin signaling inhibitors including DKK1, and these miRs are induced by Wnt/β-catenin signaling-dependent transcription [50]. Such crosstalk with miR-218 might be implicated in RA-FLS osteogenesis.

## Conclusions

In conclusion, our study showed that the expression of miR-218 was altered during the osteogenic differentiation of RA-FLS, and that miR-218 promoted the osteogenic differentiation of RA-FLS by targeting ROBO1 and suppressing DKK1. The induction of the osteogenic differentiation of proliferated FLS in RA synovial tissue has two potential effects; the attenuation of RA disease progression derived from FLS as effector cells, and the repair of destruction of bone. Therefore, strategies to provide miR-218 to RA-FLS or to boost the cellular reservoir of miR-218 might become a therapeutic strategy for RA. This attractive hypothesis should be further tested in animal models. At the least, overexpression of miR-218 might contribute to bone repair and suppression of bone erosion by the inhibition of DKK1 secretion, which we observed herein as an effect of miR-218 in RA-FLS, and modification of the inflammatory and invasive phenotype of RA-FLS.

## Additional files

Additional file 1: **Figure S1.** Alteration of miRNA expression after osteogenic induction as shown by microarray analysis. (A) There are 24 microRNAs in the downregulated group. (B) There are 12 microRNAs in the upregulated group. Values are given as means of three pairs (cultured in osteogenic induction medium vs in control medium). We defined “altered miRNA” that were upregulated or downregulated in all three pairs. (TIF 1935 kb)

Additional file 2: **Figure S2.** Pro-inflammatory cytokines modulated the expression of microRNA-218 (miR-218). RA-FLS (*n* = 4–6) were stimulated with recombinant tumor necrosis factor-α (TNF-α) (10 ng/ml) and interleukin-1β (IL-1 β) (1 ng/ml) or interleukin 6 (IL-6) (100 ng/ml) with soluble IL-6 receptor (sIL-6R) (100 ng/ml) for 24 h to explore the effects on miR-218 expression. Expression of miR-218 determined by TaqMan-based real-time polymerase chain reaction was expressed relative to the control, which was defined as 1. Values are presented as means ± SEM. (TIF 299 kb)

## Abbreviations

ACR: American College of Rheumatology
ALP: Alkaline phosphatase
BM: Bone marrow
CDH11: Cadherin 11
Ct: Comparative threshold cycle
CTNNB1: Catenin beta 1
DKK1: Dickkopf-1
DMEM: Dulbecco’s modified Eagle’s medium
ELISA: Enzyme-linked immunosorbent assay
EULAR: European League Against Rheumatism
FBS: Fetal bovine serum
FLS: Fibroblast-like synovial cells
GAPDH: Glyceraldehyde-3-phosphate dehydrogenase
IL-1β: Interleukin-1β
IL-6: Interleukin-6
miR-218: miR-218-5p
miRNA: MicroRNA
MSCs: Mesenchymal cells
NF-κB: Nuclear factor-κB
PCR: Polymerase chain reaction
pre-miR: Precursor miRNA
RA: Rheumatoid arthritis
RNU6B: U6B small nuclear RNA
ROBO1: Roundabout 1
RUNX2: Runt related transcription factor 2
SEM: Standard error of the mean
siRNA: Small interfering RNA
TNF: Tumor necrosis factor
UTR: Untranslated region
