# Supplementary figures and images for: Osteogenic differentiation of fibroblast-like synovial cells in rheumatoid arthritis is induced by microRNA-218 through a ROBO/Slit pathway
Source: Arthritis Res Ther. 2018 Aug 29;20:189. doi: 10.1186/s13075-018-1703-z (PMC6116572; doi:10.1186/s13075-018-1703-z)

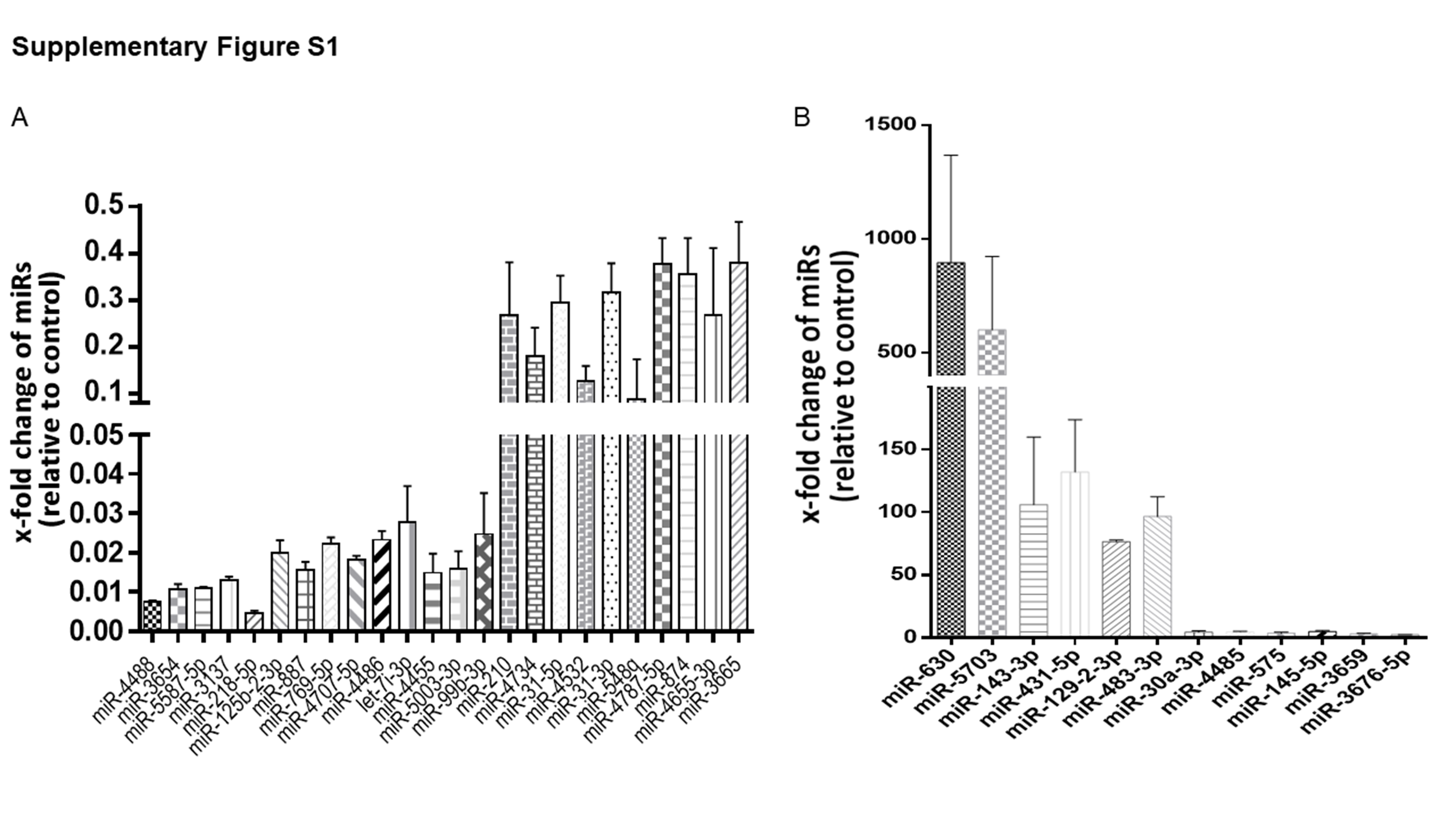

Supplement: Supplementary file 1 — Figure S1. Alteration of miRNA expression after osteogenic induction as shown by microarray analysis. (A) There are 24 microRNAs in the downregulated group. (B) There are 12 microRNAs in the upregulated group. Values are given as means of three pairs (cultured in osteogenic induction medium vs in control medium). We defined “altered miRNA” that were upregulated or downregulated in all three pairs. (TIF 1935 kb) [file 13075_2018_1703_MOESM1_ESM.tif]

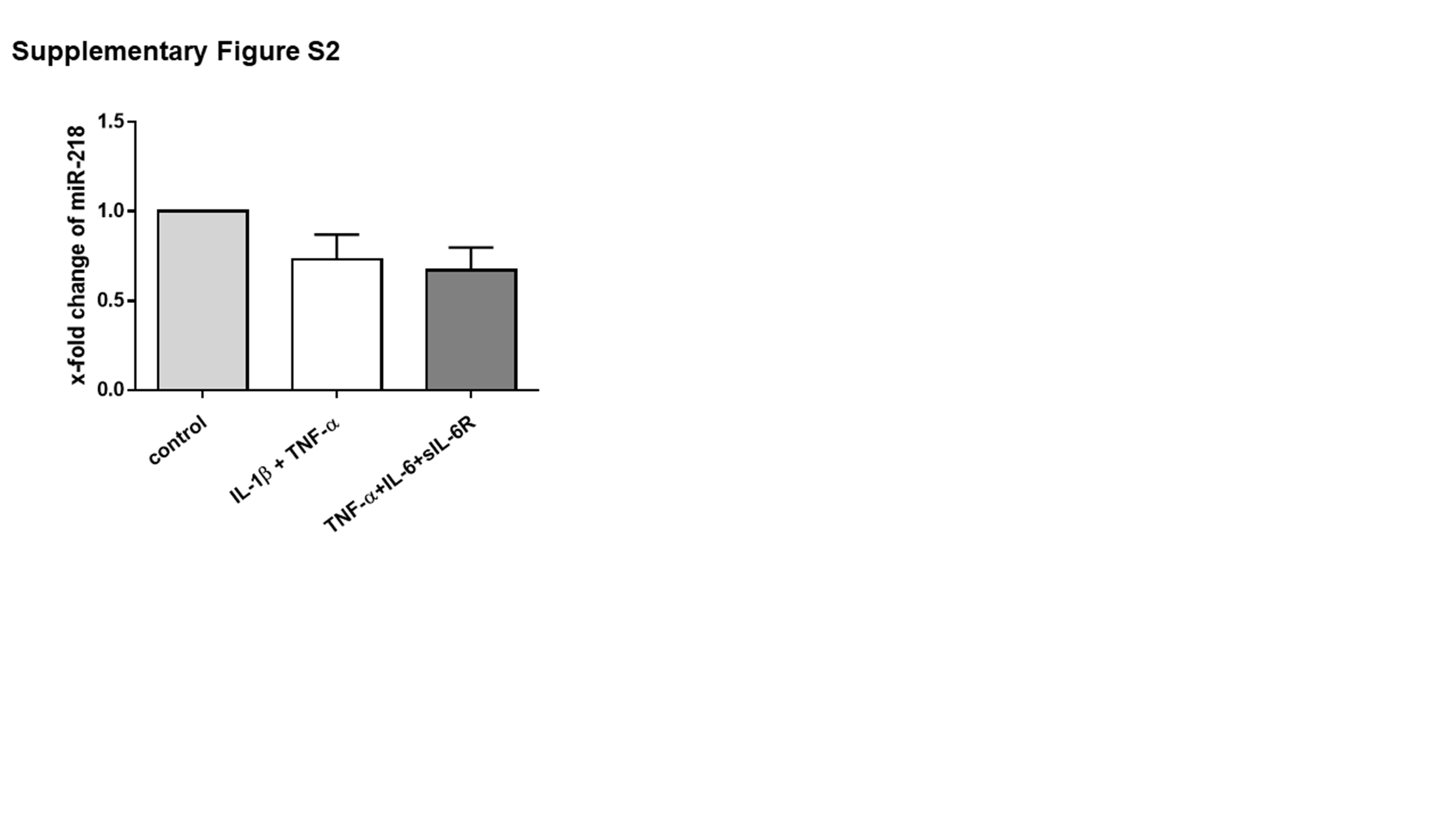

Supplement: Supplementary file 2 — Figure S2. Pro-inflammatory cytokines modulated the expression of microRNA-218 (miR-218). RA-FLS (n = 4–6) were stimulated with recombinant tumor necrosis factor-α (TNF-α) (10 ng/ml) and interleukin-1β (IL-1 β) (1 ng/ml) or interleukin 6 (IL-6) (100 ng/ml) with soluble IL-6 receptor (sIL-6R) (100 ng/ml) for 24 h to explore the effects on miR-218 expression. Expression of miR-218 determined by TaqMan-based real-time polymerase chain reaction was expressed relative to the control, which was defined as 1. Values are presented as means ± SEM. (TIF 299 kb) [file 13075_2018_1703_MOESM2_ESM.tif]
